# In Silico Drug Repurposing for Anti-Inflammatory Therapy: Virtual Search for Dual Inhibitors of Caspase-1 and TNF-Alpha

**DOI:** 10.3390/biom11121832

**Published:** 2021-12-04

**Authors:** Alejandro Speck-Planche, Valeria V. Kleandrova, Marcus T. Scotti

**Affiliations:** 1 Postgraduate Program in Natural and Synthetic Bioactive Products, Federal University of Paraíba, João Pessoa 58051-900, Brazil; mtscotti@gmail.com; 2 Laboratory of Fundamental and Applied Research of Quality and Technology of Food Production, Moscow State University of Food Production, Volokolamskoe shosse 11, 125080 Moscow, Russia; valeria.kleandrova@gmail.com

**Keywords:** anti-inflammatory, caspase-1, COVID-19, cytokine storm, drug repurposing, MLP, QSAR, TNF-alpha, virtual screening

## Abstract

Inflammation involves a complex biological response of the body tissues to damaging stimuli. When dysregulated, inflammation led by biomolecular mediators such as caspase-1 and tumor necrosis factor-alpha (TNF-alpha) can play a detrimental role in the progression of different medical conditions such as cancer, neurological disorders, autoimmune diseases, and cytokine storms caused by viral infections such as COVID-19. Computational approaches can accelerate the search for dual-target drugs able to simultaneously inhibit the aforementioned proteins, enabling the discovery of wide-spectrum anti-inflammatory agents. This work reports the first multicondition model based on quantitative structure–activity relationships and a multilayer perceptron neural network (mtc-QSAR-MLP) for the virtual screening of agency-regulated chemicals as versatile anti-inflammatory therapeutics. The mtc-QSAR-MLP model displayed accuracy higher than 88%, and was interpreted from a physicochemical and structural point of view. When using the mtc-QSAR-MLP model as a virtual screening tool, we could identify several agency-regulated chemicals as dual inhibitors of caspase-1 and TNF-alpha, and the experimental information later retrieved from the scientific literature converged with our computational results. This study supports the capabilities of our mtc-QSAR-MLP model in anti-inflammatory therapy with direct applications to current health issues such as the COVID-19 pandemic.

## 1. Introduction

Inflammation is a complex biological process in which the immune system responds to noxious stimuli caused by pathogens, damaged cells, toxic compounds, or irradiation [1,2]. The purpose of inflammation as a defense mechanism vital for health is to act by removing such harmful stimuli and initiating the healing process [3]. However, with the accelerated advancements of science and technology in the context of epidemiological, diagnostic, and clinical studies, it has become more evident that dysregulated inflammation leads to chronic medical conditions [4,5], which include (but are not limited to) cancer, autoimmune diseases (e.g., rheumatoid arthritis, diabetes mellitus type 1, psoriasis, rheumatoid arthritis, and systemic lupus erythematosus), arteriosclerosis, neurodegenerative disorders, illnesses of the liver and the kidneys, and hybrid complex diseases such as multiple sclerosis.

At the biomolecular level, caspase-1 and tumor necrosis factor-alpha (TNF-alpha) are two key proteins involved in the development and progression of dysregulated inflammation. From one side, caspase-1 acts as an inflammatory response initiator, inducing a proinflammatory response by cleaving (and thus activating) two inflammatory cytokines [6,7,8] known as interleukin 1β (IL-1β) and interleukin 18 (IL-18) while inducing pyroptosis (a programmed lytic cell-death pathway) [9,10] through the cleavage of the protein gasdermin D. On the other hand, TNF-alpha is a cytokine that has been identified as a central player in the pathogenesis of inflammation and autoimmune diseases [11] and can trigger several inflammation-related proteins such as caspase-1 [12], as well as other cytokines and chemokines [13]. An important aspect to highlight here is that both caspase-1 and TNF-alpha are essential in the development of the hyperinflammatory physiological reaction known as cytokine storm, which is present in viral infections caused by coronaviruses [14,15], including the causal agent of the current COVID-19 pandemic (SARS-CoV-2) [14,15,16]. All this suggests that anti-inflammatory therapies based on dual inhibition of caspase-1 and TNF-alpha could be an efficient option to treat a wide spectrum of inflammation-based diseases.

The drug repurposing paradigm, which relies on finding new applications for “old” drugs [17], can guide the discovery of versatile anti-inflammatory agents where in silico methods could be essential to accelerate this process. However, despite the great potential of in silico methods such as molecular docking, quantitative structure–activity relationships (QSAR), tridimensional QSAR (3D-QSAR), pharmacophore modeling, molecular dynamics, and network pharmacology, they have only been applied to the search for inhibitors of either caspase-1 [18,19,20,21,22] or TNF-alpha [18,23,24,25,26,27], never both proteins. This demonstrates the limitations of these in silico methods, which are a reflection of the current’ single-target therapies to treat inflammation-based diseases. In addition, most of these computational methods have focused on a series of structurally related molecules while relying on only one assay protocol, and some of them have not provided enough information regarding the physicochemical properties and structural requirements that are necessary for the inhibition of caspase-1 and/or TNF-alpha.

In recent years, the methodology known as perturbation theory combined with machine learning (PTML) has been reported, overcoming the aforementioned drawbacks of the current in silico methods. This comes from the fact that the PTML models can integrate different kinds of chemical and biological data, and in doing so, they can simultaneously predict multiple biological endpoints (activity, toxicity, and/or pharmacokinetic properties) against many different targets (e.g., proteins, microorganisms, cell lines, laboratory animals, and/or humans), and by considering diverse assay protocols. As a result, PTML models have had great success in therapeutic areas such as infectious diseases [28,29,30,31,32,33,34,35], cancer [36,37,38,39], and neurological disorders [40,41,42,43].

Considering all the previous ideas, in this work, we have applied the PTML modeling methodology by building a multicondition QSAR model based on a multilayer perceptron network (mtc-QSAR-MLP) to search for anti-inflammatory drugs able to act as dual inhibitors of caspase-1 and TNF-alpha. In doing so, we interpreted the mtc-QSAR-MLP model at the physicochemical and structural levels. Then, we performed in silico drug repurposing (virtual screening) of agency-regulated chemicals, i.e., molecules with known in vivo safety data and for which a controlled use exists according to the guidelines established by regulatory bodies (government authorities). Examples of agency-regulated chemicals predicted by our mtc-QSAR-MLP model included (but were not limited to) investigational and FDA-approved drugs, food components, and hazardous compounds. Some of those chemicals were identified as versatile anti-inflammatory agents via simultaneous inhibition of caspase-1 and TNF-alpha.

## 2. Materials and Methods

### 2.1. Characteristics of the Dataset and Calculation of the Molecular Descriptors

We retrieved all the chemical and inhibition data on caspase-1 and TNF-alpha from the public database known as ChEMBL [44]. The activity data were expressed as the half-maximal inhibitory concentration in nanomolar [IC_50_ (nM)]. During the curation of our dataset, we eliminated row entries for which SMILES (Simplified Molecular Input Line Entry System) codes, activity values, and/or measurement units were missing. When different IC_50_ values for the same chemical tested more than one time under the same experimental condition were found, we kept only the row entry corresponding to the lowest IC_50_ value. The dataset contained 1444 molecules, but because most of them were tested only against at least one of the two aforementioned target proteins (*tg*) and by considering at least one out of five types of experimental information (*ei*) related to different assay protocols, the dataset ended up having 1476 cases. Each combination of the elements *tg* and *ei* represents a unique experimental condition *cj*, which can be expressed as *cj*(*tg*, *ei*). In this context, each case/molecule in the dataset was labeled as active [*ACTi*(*cj*) = 1] or inactive [*ACTi*(*cj*) = −1], *ACTi*(*cj*) being a categorical variable that designated the inhibitory activity of the *i*th case/molecule under the experimental condition *cj*. Such an annotation was carried out according to the IC_50_ cutoff values that are depicted in Table 1 together with the eight different experimental conditions *cj* reported in our dataset.

We would like to highlight that the selected cutoff values reported in Table 1 prevent the excessive imbalance between the number of molecules labeled as active and the number of molecules annotated as inactive. Such cutoffs are in the very low micromolar range, while inhibition cutoff values to search for hits by using powerful experimental techniques such as high-throughput screening (HTS) start at around 10 µM [45]. Consequently, such a selection of the IC_50_ cutoff values should make our mtc-QSAR-MLP model more rigorous when searching for inhibitors of caspase-1 and TNF-alpha.

The SMILES codes belonging to the 1476 cases/molecules were stored in a txt file, which was then used as the input for the software MODESLAB v1.5 [46,47] to calculate four sets of molecular descriptors called topological indices. The first set was based on the vertex (atom) connectivity indices [*X*(*t*)*m*] [48,49]:

(1) $$X\left(t\right)m=\overset{Ns}{\underset{q=1}{\sum }}\overset{m+1}{\underset{a=1}{\prod }}{\left(\delta_{a}\right)}_{q}^{-0.5}\;$$

In Equation (1), *t* is the type of subgraph/fragment, for instance, path (P), cluster (C), path–cluster (PC), and chains/rings (Ch). Additionally, *m* is the order of the subgraph (number of bonds) while *Ns* is the number of subgraphs of type *t* and order *m*. Here, $\delta_{a}$ is the vertex (atom) degree expressed as the number of nonhydrogen atoms attached to *a*th atom.

The second set of topological indices was focused on the vertex (atom)-based valence connectivity indices [*Xv*(*t*)*m*] [49,50]:

(2) $$Xv\left(t\right)m=\overset{Ns}{\underset{q=1}{\sum }}\overset{m+1}{\underset{a=1}{\prod }}{\left(\delta_{a}^{v}\right)}_{q}^{-0.5}\;$$

where $\delta_{a}^{v}$ refers to the vertex (atom) valence degree and is calculated as:

(3) $$\delta_{a}^{v}=\frac{\left(Z_{a}^{v}-h_{a}\right)}{\left(Z_{a}-Z_{a}^{v}-1\right)}\;$$

Notice that in Equation (2), the symbols *a*, *m*, *q*, *t*, and *Ns*, have the same meanings as in Equation (1). Additionally, in Equation (3), $Z_{a}^{v}$ is the number of valence electrons of any vertex/atom *a*, $h_{a}$ is the number of hydrogen atoms bonded to the vertex/atom *a*, and $Z_{a}$ is the atomic number of that same atom. The same mathematical operation is used for all the atoms in a molecule.

We calculated the third group of topological indices, named edge (bond) connectivity indices *e*(*t*)*m* [51]:

(4) $$e\left(t\right)m=\overset{Ns}{\underset{q=1}{\sum }}\overset{m}{\underset{p=1}{\prod }}{\left[\delta {\left(e\right)}_{p}\right]}_{q}^{-0.5}\;$$

where edge (bond) degree $\delta {\left(e\right)}_{p}$ of the *p*th edge/bond is calculated as:

(5) $$\delta {\left(e\right)}_{p}=\delta_{a}+\delta_{b}\;$$

In Equation (4), the terms *m*, *q*, *t*, and *Ns* have been previously defined, while in Equation (5), $\delta_{a}$ and $\delta_{b}$ are the vertex (atom) degrees of the atoms *a* and *b*, respectively (see Equation (1)).

The last set of topological indices was based on the spectral moments of the edge (bond) adjacency matrix $SM\left(w_{a,b}\right)k$ [52,53,54]:

(6) SM(wa,b)k=Tr(Ek)=∑[e(wa,b)]k 

where $w_{a,b}$ represent the bond weight (physicochemical property) of a covalent bond between atoms *a* and *b*:

(7) $$w_{a,b}=\frac{PP_{a}}{\delta_{a}}+\frac{PP_{b}}{\delta_{b}}$$

In Equation (6), **Tr** is the trace of the edge (bond) adjacency matrix **E** and [e(wa,b)]k are the diagonal entries of the *k*th power of **E**. On the other hand, in Equation (7), $PP_{a}$ and $PP_{b}$ are the respective atomic physicochemical properties of any two atoms *a* and *b*. The atomic physicochemical properties used in this work were hydrophobicity (*Hyd*), polar surface area (*Psa*), refractivity (*Mol*), atomic weight (*Ato*), and the capacity to act as a hydrogen bond donor (*Ab-sumB2H*). The terms $\delta_{a}$ and $\delta_{b}$ are the vertex (atom) degrees of the aforementioned atoms; the definition of vertex (atom) degree has been given with Equation (1).

We also calculated size-independent topological indices:

(8) $$NTI=\frac{TI}{nB}\;$$

In Equation (8), *NTI* represents a normalized topological index and *TI* refers to any of the topological indices mentioned in Equations (1)–(6) while *nB* is the number of bonds (without counting bond multiplicity) of a molecule.

Notice that neither *TI* nor *NTI* can discriminate the effect of the chemical structure of a molecule if the experimental condition *cj* changes (e.g., different targets (*tg*) or assay protocols (*ei*)). Therefore, we calculated a series of topological indices that fused chemical and biological information. To do so, we employed an adaptation of the Box-Jenkins approach, which is the key of the PTML modeling philosophy and for which great successful applications in different research areas have been reported in the scientific literature [28,29,30,31,32,33,34,35,36,37,38,39,40,41,42,43,55,56,57]. In the first step of this approach, we used the following mathematical formula:

(9) $$avg\left[GTI\right]c_{j}=\frac{1}{n\left(cj\right)}\times \overset{n\left(cj\right)}{\underset{i=1}{\sum }}GTI_{i}\;$$

In Equation (9), *GTI* is a general symbol to represent either *TI* or *NTI*. Additionally, *n*(*cj*) is the number of cases/molecules annotated as active in the training set which were experimentally tested by considering a defined element of the experimental condition *cj*. For instance, if *n*(*cj*) = *n*(*tg*), then we are considering the number of cases/molecules labeled as active which were tested against the same target protein (*tg*). The same equation was applied to the element *ei*, meaning that *n*(*ei*) was the number of cases/molecules annotated as active which were tested by considering the same experimental information regarding the assay protocol. Consequently, *avg*[*GTI*]*cj* is an average of the *GTI* values. In the second step of the Box–Jenkins approach:

(10) $$D\left[GTI\right]cj=\left(\frac{GTI-avg\left[GTI\right]cj}{SDev\left(GTI\right)}\right)\cdot \sqrt{p\left(cj\right)}\;$$

In Equation (10), the terms *GTI* and *avg*[*GTI*]*cj* have already been defined (see Equation (9)). The term *p*(*cj*) is the *a priori* probability of finding a case/molecule tested by considering a defined element of the experimental condition *cj*. Here, *p*(*cj*) is calculated as the ratio of *n*(*cj*) to *Nt* (number of cases/molecules present in the training set) and, as in the case of *n*(*cj*), its definition was applied separately to each of the elements of the experimental condition *cj* (*tg* and *ei*). On the other hand, *SDev*(*GTI*) is the standard deviation calculated from the *GTI* values (considering only the training set), and *D*[*GTI*]*cj* is a descriptor that considers both the chemical structure of a molecule and the element of the experimental condition *cj* (*tg* or *ei*) under which a molecule was assayed. We would like to emphasize that the joint interpretation of Equations (9) and (10) indicates that by using the *D*[*GTI*]*cj* descriptors as inputs, one can create a PTML model able to predict the inhibitory activity of any molecule as many times as experimental conditions *cj* (combinations of the elements *tg* and *ei*). Particularly, as depicted in Table 1, the purpose here was to build an mtc-QSAR-MLP model to predict the inhibitory activity of a molecule under eight different experimental conditions. As mentioned in the previous section, such a capability of simultaneously predicting complex biological endpoints under dissimilar experimental conditions is an intrinsic characteristic of any PTML model [28,29,30,31,32,33,34,35,36,37,38,39,40,41,42,43,55,56,57].

### 2.2. Development of the Mtc-QSAR-MLP Model

**Figure 1:**
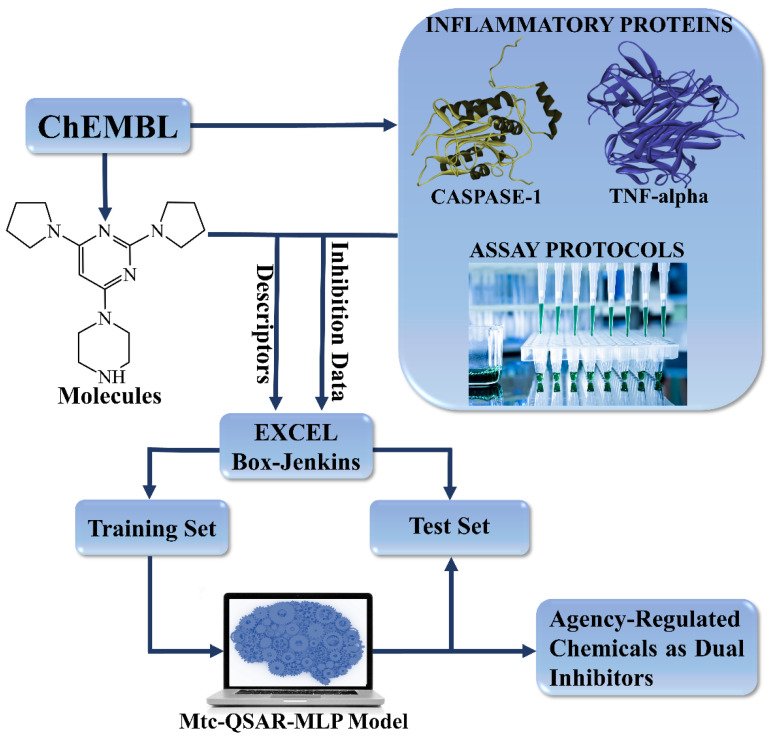
Development and application of an mtc-QSAR-MLP model.

To generate the mtc-QSAR-MLP model, we followed a series of guidelines that are depicted in Figure 1.

The 1476 cases/molecules present in our dataset appear ordered according to their increasing IC_50_ values. We randomly split them into training and test series [28,29,30,31,32,33,34,35,36,37,38,39,40,41,42,43,55,56,57] in such a way that the first three out of four cases/molecules were annotated to belong to the training set while the fourth was considered to belong to the test set. This procedure was repeated for the entire dataset. Therefore, the training set was used to search for the best mtc-QSAR-MLP model and contained 1111 molecule/cases (75.27% of our dataset), 464 active and 647 inactive. The test set, which was employed to confirm the predictive power of the mtc-QSAR-MLP model, comprised 365 molecules/cases (24.73% of the dataset), 153 active and 212 inactive.

In our dataset, before using the *D*[*GTI*]*cj* descriptors as inputs to build the mtc-QSAR-MLP model, we computed the metric known as the information gain ratio (IGR) [58] to rank the *D*[*GTI*]*cj* descriptors in terms of their potential influence/discriminatory power. To accomplish this task, the software IMMAN v1.0 [59] was employed. While selecting the most significant *D*[*GTI*]*cj* descriptors (highest IGR values), we examined the correlations among them through the pairwise Pearson correlation coefficient (*PCC*) [60]; we selected those *D*[*GTI*]*cj* descriptors with −0.6 < *PCC* < 0.6 to avoid information redundancy as much as possible. Notice that the present cutoff interval chosen for *PCC* was derived not only from already established statistical criteria [61,62,63] but also from our experience in working with topological indices.

Our mtc-QSAR-MLP model was based on an artificial neural network with a multilayer perceptron (MLP) architecture. The reason to directly use the MLP networks was based on the fact that, in addition to their versatility to solve complex tasks, MLP networks have been widely used in QSAR modeling [64,65,66]. On the other hand, MLP networks have proven to be very effective in modeling extremely complex problems in the context of the PTML philosophy [29,36,67,68,69,70]. In the process of choosing the best MLP networks (mtc-QSAR-MLP model), we considered different global statistical indices such as sensitivity (*Sn*(%)—the percentage of cases/molecules correctly classified as active), specificity (*Sp*(%)—the percentage of cases/molecules correctly classified as inactive), accuracy (*Acc*(%)—the percentage of correctly classified cases/molecules considering both active and inactive), and the Matthews’ correlation coefficient (*MCC*) [71]. In any case, the selection of the best mtc-QSAR-MLP model was based on the analysis of the values of the local sensitivities (*Sn*(%))*tg* and (*Sn*(%))*ei* as well as the local specificities (*Sp*(%))*tg* and (*Sp*(%))*ei*. The values of these local metrics were expected to be as high as possible. The computer program STATISTICA v13.5.0.17 (Palo Alto, CA, USA) [72] was used to analyze different MLP networks in terms of the aforementioned metrics, which led to the finding of the mtc-QSAR-MLP model.

### 2.3. Assessing the Applicability Domain

We determined the applicability domain (AD) of the mtc-QSAR-MLP model according to the bounding box (descriptors’ space) approach by using a modification reported by Speck-Planche [73]. In this sense, local scores of applicability domain for each *D*[*GTI*]*cj* descriptor were calculated (LSAD_*D*[*GTI*]*cj*). If for defined case/molecule, its value of a given *D*[*GTI*]*cj* descriptor was between the maximum and minimum *D*[*GTI*]*cj* values, then, the corresponding LSAD_*D*[*GTI*]*cj* was equal to one; otherwise, the LSAD_ *D*[*GTI*]*cj* was zero. This analysis was carried out for each *D*[*GTI*]*cj* descriptor present in the mtc-QSAR-MLP model. Then, the total score of applicability domain (TSAD) for that case/molecule was calculated as the sum of all the LSAD_ *D*[*GTI*]*cj* values. When the TSAD value for the aforementioned query molecule was equal to the number of *D*[*GTI*]*cj* descriptors present in the mtc-QSAR-MLP model, the query molecule was set to belong to the AD of the mtc-QSAR-MLP model; otherwise, the query molecule was outside the AD.

### 2.4. Molecular Descriptors and their Physicochemical and Structural Meanings

When interpreting the *D*[*GTI*]*cj* descriptors, we considered their relative significances in the model measured by the sensitivity values (*SV*), which have been used to rank molecular descriptors before [74]. In doing so, we qualitatively estimated the tendency of variation of each in the mtc-QSAR-MLP model. This means that we could describe how the distribution of the different atoms through their physicochemical property varied (decreased or increased) the probability of a molecule to be active against the desired targets [73,75], in this particular case, caspase-1 and TNF-alpha. In addition, we mentioned a series of key molecular fragments whose presence could confer dual activity to a molecule against caspase-1 and TNF-alpha.

### 2.5. Virtual Predictions of Single- and Dual-Target Inhibitors

For the case of the single-target inhibitors, to demonstrate the efficiency of our mtc-QSAR-MLP model, we discussed different molecules in both our dataset (training and test sets) and a virtual screening database containing 8922 chemicals reported by different regulatory agencies (e.g., FDA). Such molecules were experimentally reported in ChEMBL and we examined whether our mtc-QSAR-MLP model converged with the experimental results. When searching for dual-target inhibitors which is the main purpose of this work, we used a series of metrics recently reported by Speck-Planche and coworkers. Such metrics were FA(%) and S(TSAD) [76]. For a query molecule, FA(%) measured the frequency (percentage of times) in which a molecule was predicted as active by considering the eight experimental conditions *cj* reported in this work (see Table 1). On the other hand, for the same query molecule, S(TSAD) was obtained as the sum of all the TSAD values reported for that molecule by considering the eight experimental conditions *cj*. Consequently, S(TSAD) was a general score given to a query molecule which permitted us to know whether the molecule was within the AD of the mtc-QSAR-MLP model by considering the predictions performed under the eight experimental conditions *cj*.

## 3. Results and Discussion

### 3.1. The Mtc-QSAR-MLP Model: Performance and Applicability Domain

By considering all the statistical metrics mentioned above, the most appropriate mtc-QSAR-MLP model found by us has the profile MLP 11-38-2, which means that 11 *D*[*GTI*]*cj* descriptors (Table 2) were used as input nodes (input layer), 38 neurons were present in the hidden layer, and two possible values of the categorical variable *ACTi*(*cj*) were predicted in the output layer: active [*ACTi*(*cj*) = 1] and inactive [*ACTi*(*cj*) = −1].

In terms of performance, in the training set, the mtc-QSAR-MLP model could correctly classify 417 out of 464 active and 610 out of 647 inactive cases/molecules, which was equivalent to *Sn*(%) = 89.87% and *Sp*(%) = 94.28%. The metric *Acc*(%) achieved a value of 92.44%. In the case of the test set, the mtc-QSAR-MLP model rightly classified 133 out of 153 active [*Sn*(%) = 86.93%] and 190 out of 212 inactive [*Sp*(%) = 89.62%] cases/molecules, which translated into *Acc*(%) = 88.49%. In addition, the metric *MCC* exhibited values of 0.844 and 0.764 for training and test sets, respectively. The closeness of these *MCC* values to one indicates the strong correlation/convergence between the observed (*ACTi*(*cj*)) and the predicted (*Pred*[*ACTi*(*cj*)]) categorical values of inhibitory activity. All the chemical and inhibition data are present in Supplementary Material S1 while the classification/prediction results for each case/molecule in our dataset can be found in Supplementary Material S2.

If now we consider the values of the local sensitivities and specificities (also reported in Supplementary Material S2), we will see that for the case of target proteins (*tg*), in the training set, [*Sn*(%)]*tg* > 87% and [*Sp*(%)]*tg* > 92%. This means that in the training set, for both caspase-1 and TNF-alpha, the mtc-QSAR-MLP model could correctly classify at least 87% and 92% of active and inactive cases/molecules. In the test set, [*Sn*(%)]*tg* > 83% and [*Sp*(%)]*tg* > 87% were achieved. The same deductions made for the training set regarding the percentages of correctly classified active and inactive cases/molecules can also be applied to the test set. Regarding the experimental information associated with the different assay protocols (*ei*), in the training set, [*Sn*(%)]*ei* was in the interval 75–91.74% and [*Sp*(%)]*ei* exhibited the range 88.36–100%, clearly demonstrating that, by considering the different assay protocols, at least 75% of active and 88.36% of inactive were rightly classified by the mtc-QSAR-MLP model. In the test set, a similar behavior was observed since [*Sn*(%)]*ei* and [*Sp*(%)]*ei* spanned 76.92–89.74% and 80.00–100%, respectively. The only exception was the value of [*Sn*(%)]*ei* = 0 reported for the assay protocol labeled as “F (cell-based format)” for which only one active compound was experimentally tested, and therefore is not enough to estimate the performance of our mtc-QSAR-MLP model in that particular assay protocol. The joint analysis of the global and the local statistical indices mentioned above confirms the quality and predictive power of the mtc-QSAR-MLP model to classify/predict chemicals against caspase-1 and TNF-alpha under dissimilar experimental conditions.

Regarding the estimation of the AD (Supplementary Material S3), we should recall that there are 11 *D*[*GTI*]*cj* descriptors present in the mtc-QSAR-MLP model, which means that only the cases/molecules for which the metric TSAD (see Section 2.3) is equal to 11 will be within the AD of the mtc-QSAR-MLP model. In our dataset, only seven cases/molecules were outside the AD, five of them with TSAD = 10 and the other two with TSAD = 9. In any case, we kept these seven cases/molecules because their removal did not improve the mtc-QSAR-MLP model. In addition, all these seven cases/molecules were correctly classified by the mtc-QSAR-MLP model.

### 3.2. Physicochemical and Structural Interpretation of the Molecular Descriptors

**Figure 2:**
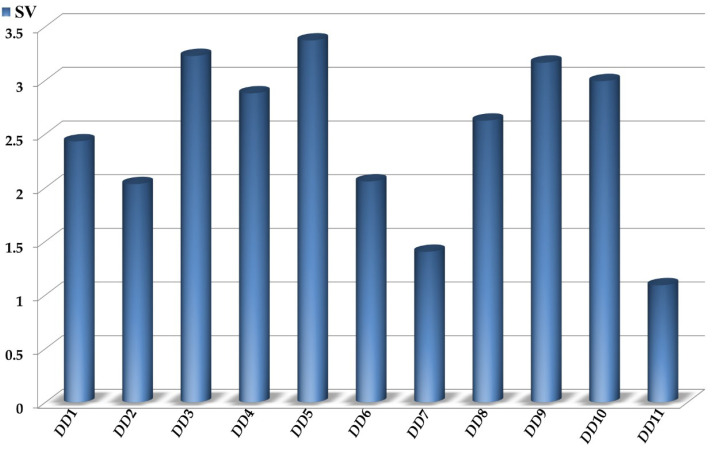
Relative importance of the *D*[*GTI*]*cj* descriptors in the mtc-QSAR-MLP model. The following abbreviations are used: *DD*1 = *D*[*Xv*(*PC*)6]*tg*, *DD*2 = *D*[*Xv*(*Ch*)6]*tg*, *DD*3 = *D*[*e*(*Ch*)5]*tg*, *DD*4 = *D*[*NSM*(*Hyd*)3]*tg*, *DD*5 = *D*[*NX*(*P*)6]*tg*, *DD*6 = *D*[*Ne*(*P*)2]*tg*, *DD*7 = *D*[*SM*(*Psa*)7]*ei*, *DD*8 = *D*[*e*(*C*)4]*ei*, *DD*9 = *D*[*NSM*(*Mol*)1]*ei*, *DD*10 = *D*[*NSM*(*Ato*)1]*ei*, and *DD*11 = *D*[*NSM*(*Ato*)5]*ei*.

As mentioned in Section 2.4, when interpreting the *D*[*GTI*]*cj* descriptors in the mtc-QSAR-MLP model, the *SVs* (Figure 2) allowed us to identify the most influential.

Additionally, Table 3 contains two means calculated (using only the training set) for each of the *D*[*GTI*]*cj* descriptors: one considering only active cases/molecules and the other based on the inactive ones [73,75,76]. The tendency of variation reported in Table 3 gives a fast insight regarding how the increase or decrease of the value of each *D*[*GTI*]*cj* descriptor can improve the probability of a molecule to be active against both caspase-1 and TNF-alpha.

We would like to emphasize that the *D*[*GTI*]*cj* descriptors are directly derived from their corresponding counterparts calculated by using Equations (1)–(8), and therefore, from a physicochemical and structural point of view, the *D*[*GTI*]*cj* descriptors contain the same information. That being said, we can see that one of the most important properties described by our mtc-QSAR-MLP model is the molecular accessibility since this is accounted for by 3 of the 11 *D*[*GTI*]*cj* descriptors (see Figure 2 and Table 3): *D*[*Xv*(*PC*)6]*tg*, *D*[*Xv*(*Ch*)6]*tg*, and *D*[*NX*(*P*)6]*tg*. These are derived from the atom-based connectivity indices, which have been mathematically proven as measures of the molecular accessibility [77,78]. Notice that the molecular accessibility can either measure the propensity of different regions of a molecule to interact with the surrounding medium [77,78] (e.g., in our case, water molecules or amino acids within the pocket of a protein) or the ability of other regions to positively (or negatively) contribute to steric factors (e.g., fitting in the pocket of a protein).

The descriptor *D*[*Xv*(*PC*)6]*tg* involves information regarding the decrease of the molecular accessibility (steric hindrance) of fragments, where functional groups such as −CX_3_ (X = halogen), −SO_2_NHR (R = any atom), or −PZ_4_ (Z = any atom, with the four Z atoms being either equal or different) are attached to any ring (or an atom already bonded to two other atoms). Therefore, the aforementioned fragments should be avoided. In the mtc-QSAR-MLP model, *D*[*Xv*(*PC*)6]*tg* is the seventh most important. Another descriptor is *D*[*Xv*(*Ch*)6]*tg* (the ninth most significant), which characterizes the increase in the molecular accessibility in six-membered rings. Here, the number of such rings should be increased; aromatic rings are preferred over their aliphatic counterparts while heterocycles are preferred over cycles lacking heteroatoms. Additionally, the lower the substitution of a six-membered ring, the higher will be its positive influence of that ring on the dual inhibitory activity against caspase-1 and TNF-alpha. The *D*[*GTI*]*cj* descriptor with the highest significance is *D*[*NX*(*P*)6]*tg,* and it considers the increment of the molecular accessibility by increasing the number of large fragments containing at least six bonds (without counting bond multiplicity). This *D*[*GTI*]*cj* descriptor demonstrates that the binding pockets of caspase-1 and TNF-alpha are relatively large, and therefore, a molecule should be large enough to fit in the pocket of the proteins, with the subsequent occurrence of stronger ligand–protein interactions.

Another important property is the volume, which is characterized by three other *D*[*GTI*]*cj* descriptors: *D*[*e*(*Ch*)5]*tg*, *D*[*Ne*(*P*)2]*tg*, and *D*[*e*(*C*)4]*ei*. These *D*[*GTI*]*cj* descriptors conserve the same information as their counterparts, the edge (bond)-based connectivity indices, and therefore, they are measures of contribution to the molecular/molar volume of a molecule [51,79,80]. Thus, *D*[*e*(*Ch*)5]*tg*, indicates the increment of the number of five-membered rings as a way to increase the molecular volume in the sense of fitting better in the pocket of the proteins. Again, five-membered rings with low substitutions are preferred. We would like to highlight that *D*[*e*(*Ch*)5]*tg* is the second most important descriptor in the mtc-QSAR-MLP model. On the other hand, the diminution of the value of *D*[*Ne*(*P*)2]*tg* (the eighth most influential) involves the increment of fused rings in the periphery of a molecule as well as ramifications (involving fragments formed by two bonds) in the central part of that molecule, making it more spheric, and therefore, more capable of effectively interacting with the protein residues. Then, we have *D*[*e*(*C*)4]*ei*, whose decrease in value means that the presence of moieties where an atom is bonded to more than three (e.g., quaternary carbon, sulfur in sulfonic acids and derivatives, and most of the phosphorus-containing functional groups) must be avoided. This happens because such moieties considerably increase the steric hindrance and decrease the planarity of a molecule. The descriptor *D*[*e*(*C*)4]*ei* is the sixth most significant descriptor.

In the mtc-QSAR-MLP model, there are five *D*[*GTI*]*cj* descriptors derived from the so-called spectral moments of the bond adjacency matrix (see Equation (6)), which characterize the concentration of different physicochemical properties in regions of different sizes in a molecule [52,53,54,81]. Thus, *D*[*NSM*(*Hyd*)3]*tg* indicates (regardless of the size of a molecule) the diminution of the hydrophobicity in molecular fragments such as three-membered rings and functional groups where an atom is bonded to other two atoms. For this reason, the presence of functional groups such as carbonyl, carboxyl and its derivatives, moieties containing secondary alcohols, ureas, and carbamates, as well as the three-membered heterocycles (heteroatom = N or O) is favorable for the diminution of the value of *D*[*NSM*(*Hyd*)3]*tg* (having the fifth-highest significance) and the subsequent increment of the probability of a molecule to exhibit dual inhibitory activity against caspase-1 and TNF-alpha. This is because the presence of those fragments increases the propensity for the formation of hydrogen bond interactions. The presence of polar functional groups and three-membered heterocycles is further supported by *D*[*SM*(*Psa*)7]*ei* (the tenth most important descriptor), which characterizes the augmentation of the polar surface area in these molecular fragments as well as others formed by seven bonds or less, for instance, four- and five-membered heterocycles (heteroatom = N or O). Other molecular fragments such as pyrimidine-2-carboxylic acid (or the amide) will considerably increase the value of *D*[*SM*(*Psa*)7]*ei*.

On the other hand, we also have *D*[*NSM*(*Mol*)1]*ei* as an indicator of the diminution of the polarizability of a molecule, which means that nitrogen, oxygen, fluorine atoms are preferred over the others. In our mtc-QSAR-MLP model, *D*[*NSM*(*Mol*)1]*ei* is the third most important descriptor. Similarly, *D*[*NSM*(*Ato*)1]*ei* (the fourth most influential descriptor) favors the aforementioned atoms since it considers the diminution of the molecular weight, but the big difference with *D*[*NSM*(*Mol*)1]*ei* is that *D*[*NSM*(*Ato*)1]*ei* prioritizes the presence of carbon atoms over any other atom type. Lastly, *D*[*NSM*(*Ato*)5]*ei* is the least significant descriptor in the mtc-QSAR-MLP model, accounting for the diminution of the atomic weight in regions formed by five bonds or less, thus favoring the presence of linear aliphatic chains while avoiding the presence of three- and five-membered rings; if such rings are present, then they should lack heteroatoms.

### 3.3. The Mtc-QSAR-MLP Model: Virtual Analysis of Single- and Dual-Target Inhibitors

As confirmed in Section 3.1, our mtc-QSAR-MLP model exhibits very good performance in terms of correctly classifying/predicting active and inactive cases/molecules against both caspase-1 and TNF-alpha. Here, we will show that the mtc-QSAR-MLP model was able to correctly identify the privileged chemical structures belonging to drugs/chemicals that are reported as inhibitors of caspase-1 and/or TNF-alpha.

**Figure 3:**
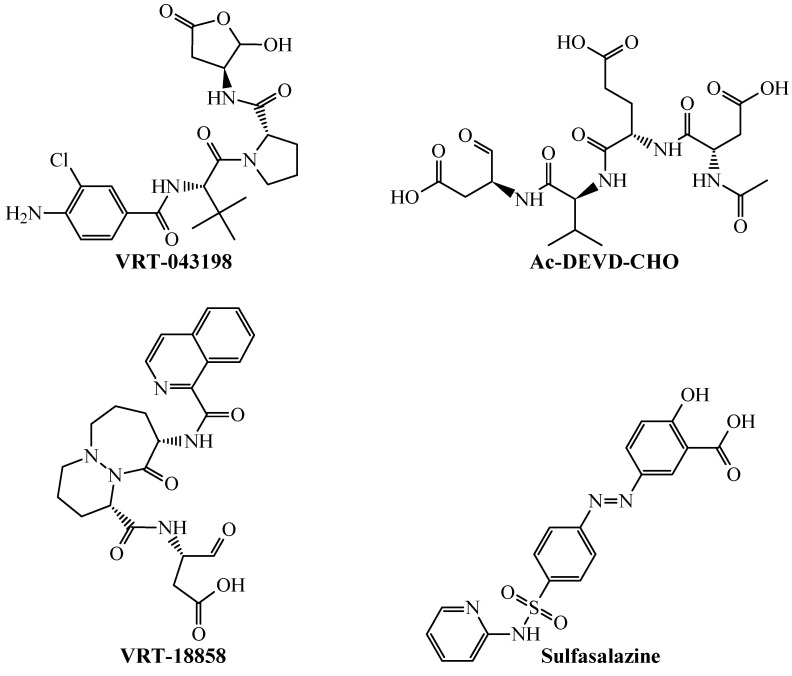
Chemical structures of molecules accurately predicted by the mtc-QSAR-MLP model as caspase-1 inhibitors.

In the sense of correctly predicting selective caspase-1 inhibitors (Figure 3), our mtc-QSAR-MLP could identify drugs such as VRT-18858, VRT-043198, and sulfasalazine, in addition to the chemical Ac-DEVD-CHO.

**Figure 4:**
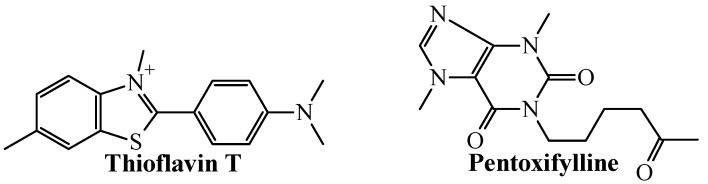
Chemicals correctly classified by the mtc-QSAR-MLP model as TNF-alpha inhibitors.

In the case of the TNF-alpha inhibitors (Figure 4), the mtc-QSAR-MLP model correctly classified/predicted the chemicals Thioflavin T and pentoxifylline.

The predictions performed by the mtc-QSAR-MLP model for all the aforementioned inhibitors can be found in Table 4.

The results of the predictions from Table 4 demonstrate several important aspects regarding the mtc-QSAR-MLP model. From one side, by inspecting the chemical structures of the different molecules in Figure 3 and Figure 4, it is clear that the mtc-QSAR-MLP model can predict chemicals belonging to a great chemical heterogenicity. On the other hand, the mtc-QSAR-MLP model is very sensitive to changes in the experimental information regarding the assay protocols. This is the case of the chemical Ac-DEVD-CHO, which, for two different assay protocols, exhibited two different IC_50_ values against caspase-1. In any case, both times, Ac-DEVD-CHO was correctly predicted by the mtc-QSAR-MLP model, in one assay protocol as active, and in the other as inactive. The predictions performed by the mtc-QSAR-MLP model for Ac-DEVD-CHO are even more compelling since this chemical was reported in one assay protocol to belong to the training set, and in the other assay protocol to be part of the test set.

We should highlight that the molecules from Table 4 belonging to the “virtual screening” set were predicted eight times because of the eight experimental conditions *cj* reported in our dataset. Therefore, their probability values are averages calculated from the predicted probabilities assessed in each of the eight experimental conditions *cj*. In the end, the great success of the mtc-QSAR-MLP model in identifying protein inhibitors is due to the high information content of the *D*[*GTI*]*cj* descriptors (see Section 3.2) in terms of the physicochemical properties and structural features that they consider. The high predicted probabilities shown in Table 4 indicate how accurately these *D*[*GTI*]*cj* descriptors characterize the chemical diversity and complexity.

The main purpose of building the present mtc-QSAR-MLP model was to use it as a tool to perform in silico drug repurposing by virtually screening large databases for the detection of potential dual inhibitors against caspase-1 and TNF-alpha (see Section 2.5). Since an external database formed by 8922 agency-regulated chemicals (Supplementary Material S4) was predicted by considering the eight experimental conditions *cj* analyzed in this work, we performed a total of 71,376 predictions (Supplementary Material S5). In this sense, Table 5 contains the top 20 ranked agency-regulated chemicals for which ideal values of FA(%) = 100% and S(TSAD) = 88 were achieved.

**Figure 5:**
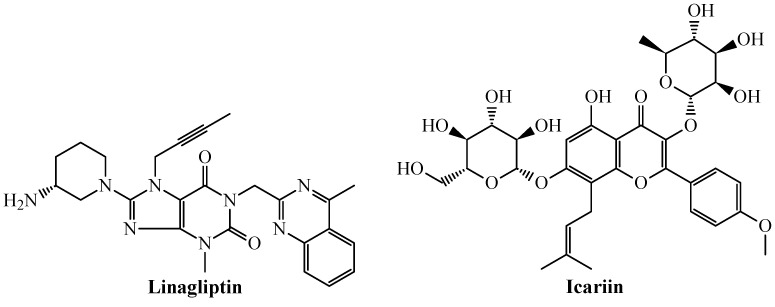
Some of the top-ranked drug/agency-regulated chemicals predicted as dual inhibitors of caspase-1 and TNF-alpha inhibitors.

We ran a search in the scientific literature using the keywords “caspase-1” and “TNF-alpha”, combining each of them with the names of the 20 agency-regulated chemicals depicted in Table 5. In doing so, we found experimental evidence on two of those chemicals (Figure 5) that suggests their potential to inhibit both caspase-1 and TNF-alpha.

First, we have linagliptin, a medication used to treat diabetes mellitus type 2. In addition to its FDA-approved use, experimental evidence indicates that the kidneys of rats injected with the drug doxorubicin underwent nephropathy showing a remarkable upregulation of the components of the NLRP3 inflammasome, including caspase-1 [82]. Such upregulation of caspase-1 was effectively suppressed by linagliptin. Regarding the inhibition of TNF-alpha, a recent study reported a quantitative sandwich enzyme immunoassay technique involving an antibody specific to quantify this protein [83]. In this sense, treatment of human U937 monocytes with linagliptin diminished the TNF-alpha levels, suggesting the anti-TNF-alpha activity of the drug [83].

The second chemical, icariin, is a prenylated flavonol glycoside, which has been studied in clinical trials for the treatment of bipolar disorder, depression, and alcohol consumption [84]. In a recent preclinical in vivo study carried out on Wistar rats used as models of osteoarthritis, icariin was demonstrated to exhibit notable anti-inflammatory effects through the inhibition (in a dose-dependent manner) of pyroptosis [85], the classical and highly inflammatory form of lytic programmed cell death that is mediated by caspase-1. In addition, icariin decreased the levels of IL-1β and IL-18, the two cytokines which are the results of protein cleavage caused by caspase-1 [85]. In another report using human keratinocytes, it was shown that icariin inhibited the inflammatory response mediated by several proteins, which included TNF-alpha [86].

**Figure 6:**
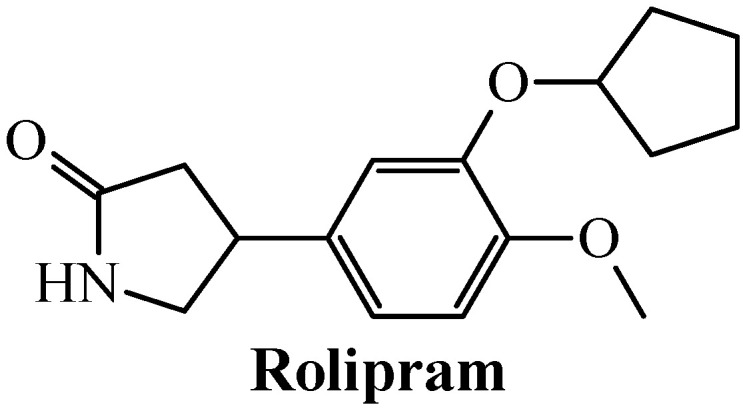
Rolipram: a promising drug computationally predicted and experimentally reported as a dual inhibitor of caspase-1 and TNF-alpha.

Altogether, the experimental results on linagliptin and icariin suggest that these two chemicals could be further studied as dual inhibitors of caspase-1 and TNF-alpha. A fast search performed on the ChEMBL database allowed us to know that linagliptin and icariin inhibit dipeptidyl peptidase 4 (DPP4) and cGMP-specific phosphodiesterase type 5 (PDE5), respectively. Therefore, we investigated if other agency-regulated chemicals acting as inhibitors of DPP-4 and PDE5 (and other proteins belonging to the same family) could also inhibit caspase-1 and TNF-alpha. By maintaining the ideal value of 88 for the metric S(TSAD) while setting FA(%) to an acceptable cutoff value of 75%, we found out that other DPP-4 inhibitors such as anagliptin [FA(%) = 87.5%] and omarigliptin [FA(%) = 75%] and PDE5 blockers such as mirodenafil [FA(%) = 75%] were also identified as dual inhibitors of caspase-1 and TNF-alpha, and therefore they should be considered for future studies. However, a surprising finding was the case of rolipram (Figure 6), a phosphodiesterase-4 (PDE4) inhibitor indicated as an antidepressant drug.

From one side, rolipram potently inhibits TNF-alpha in the nanomolar range, with IC_50_ = 100–550 nM [87,88]. At the same time, in a recent investigation involving the use of salivary acinar and ductal cells, it was confirmed that the well-established inflammasome component caspase-1 was inhibited by rolipram [89]. In the end, the remarkable inhibition of caspase-1 and TNF-alpha reported for linagliptin, icariin, and rolipram by considering in vitro and/or in vivo assays confirm that these agency-regulated chemicals can act as dual inhibitors of the aforementioned proteins.

## 4. Conclusions

The key roles of the proteins caspase-1 and TNF-alpha in inflammation make them suitable targets for a wide range of therapeutic applications. Dual inhibitors of these two proteins represent an encouraging horizon in anti-inflammatory research. The development of the present mtc-QSAR-MLP model has been an attempt to accelerate the search for wide-spectrum anti-inflammatory agents via dual inhibition of caspase-1 and TNF-alpha, which has led to the identification of three agency-regulated chemicals as versatile anti-inflammatory drugs. In this context, linagliptin, icariin, and rolipram deserve further studies, which could pave the way to the use of these chemicals for the treatment of autoimmune diseases, cancers, and even severe COVID-19 cases, since the latter medical condition is characterized by hyperinflammation (cytokine storm) where simultaneous inhibition of caspase-1 and TNF-alpha could be essential for an efficacious treatment.

## Figures and Tables

**Table 1:** Experimental conditions under which the molecules were assayed against caspase-1 and TNF-alpha.

Cutoff ^a^	*tg* ^b^	*ei* ^c^
IC_50_ ≤ 1100 nM	Caspase-1	B (assay format)
		B (single-protein format)
		B (cell-based format)
IC_50_ ≤ 1635 nM	TNF-alpha	B (single-protein format)
		F (assay format)
		B (assay format)
		B (cell-based format)
		F (cell-based format)

^a^ Value from which a molecule was considered and annotated as active [*ACTi*(*cj*) = 1] regardless of the experimental conditions under which the molecules were assayed against caspase-1 and TNF-alpha. ^b^ Protein target. ^c^ Experimental information associated with different assay protocols. Annotations combine the columns “assay type” (first letter) and “BioAssay Ontology” (phrase between parentheses). The aforementioned columns appear in any ChEMBL file containing activity data.

**Table 2:** Symbols and definitions of the *D*[*GTI*]*cj* descriptors used to build the mtc-QSAR-MLP model.

Symbol	Definition
*D*[*Xv*(*PC*)6]*tg*	Deviation of the Kier–Hall valence connectivity index of order six based on path–cluster subgraphs, depending on the chemical structure and the protein target against which each molecule was tested
*D*[*Xv*(*Ch*)6]*tg*	Deviation of the Kier–Hall valence connectivity index of order six based on chain (ring) subgraphs, depending on the chemical structure and the protein target against which each molecule was tested
*D*[*e*(*Ch*)5]*tg*	Deviation of the edge (bond) connectivity index of order five based on chain (ring) subgraphs, depending on the chemical structure and the protein target against which each molecule was tested
*D*[*NSM*(*Hyd*)3]*tg*	Deviation of the normalized edge (bond) spectral moment of order three weighted by the hydrophobicity, depending on the chemical structure and the protein target against which each molecule was tested
*D*[*NX*(*P*)6]*tg*	Deviation of the normalized Kier–Hall connectivity index of order six based on path subgraphs, depending on the chemical structure and the protein target against which each molecule was tested
*D*[*Ne*(*P*)2]*tg*	Deviation of the normalized edge (bond) connectivity index of order two based on path subgraphs, depending on the chemical structure and the protein target against which each molecule was tested
*D*[*SM*(*Psa*)7]*ei*	Deviation of the edge (bond) spectral moment of order seven weighted by the polar surface area, depending on the chemical structure and the information regarding each experimental assay
*D*[*e*(*C*)4]*ei*	Deviation of the edge (bond) connectivity index of order four based on cluster subgraphs, depending on the chemical structure and the information regarding each experimental assay
*D*[*NSM*(*Mol*)1]*ei*	Deviation of the normalized edge (bond) spectral moment of order one weighted by the molar refractivity, depending on the chemical structure and the information regarding each experimental assay
*D*[*NSM*(*Ato*)1]*ei*	Deviation of the normalized edge (bond) spectral moment of order one weighted by the atomic weight, depending on the chemical structure and the information regarding each experimental assay
*D*[*NSM*(*Ato*)5]*ei*	Deviation of the normalized edge (bond) spectral moment of order five weighted by the atomic weight, depending on the chemical structure and the information regarding each experimental assay

**Table 3:** The *D*[*GTI*]*cj* descriptors and their tendencies of variation.

Descriptors	Active Molecules	Inactive Molecules	Tendency of Variation ^a^
*D*[*Xv*(*PC*)6]*tg*	9.8432 × 10^−3^	5.7266 × 10^−2^	Decrease
*D*[*Xv*(*Ch*)6]*tg*	8.8163 × 10^−3^	−5.2871 × 10^−2^	Increase
*D*[*e*(*Ch*)5]*tg*	5.2372 × 10^−2^	−4.5470 × 10^−1^	Increase
*D*[*NSM*(*Hyd*)3]*tg*	−2.5230 × 10^−2^	3.5521 × 10^−1^	Decrease
*D*[*NX*(*P*)6]*tg*	2.1837 × 10^−2^	−1.7328 × 10^−1^	Increase
*D*[*Ne*(*P*)2]*tg*	3.2609 × 10^−3^	1.1692 × 10^−1^	Decrease
*D*[*SM*(*Psa*)7]*ei*	4.0458 × 10^−3^	−6.3587 × 10^−2^	Increase
*D*[*e*(*C*)4]*ei*	7.5688 × 10^−3^	1.0868 × 10^−1^	Decrease
*D*[*NSM*(*Mol*)1]*ei*	−1.1431 × 10^−2^	3.6017 × 10^−1^	Decrease
*D*[*NSM*(*Ato*)1]*ei*	−3.8603 × 10^−2^	5.0826 × 10^−1^	Decrease
*D*[*NSM*(*Ato*)5]*ei*	−3.0209 × 10^−3^	9.5968 × 10^−2^	Decrease

**^a^** Increase (or decrease) of the value of a *D*[*GTI*]*cj* descriptor leading to the increase in the dual inhibitory activity against caspase-1 and TNF-alpha.

**Table 4:** Different drugs or drug-derived chemicals correctly predicted by the mtc-QSAR-MLP model as protein inhibitors according to their experimental IC_50_ values reported in the ChEMBL database.

ChEMBL ID	Name	IC_50_ (nM) ^a^	*tg* ^b^	*ei* ^c^	Series ^d^	Observation ^e^	Prob.(%) ^f^
CHEMBL437105	VRT-18858	3.4	Caspase-1	B (single-protein format)	Train	Active	100
CHEMBL437105	VRT-18858	670		B (cell-based format)	Train	Active	100
CHEMBL4217577	VRT-043198	5		B (assay format)	Train	Active	80.96
CHEMBL417149	Ac-DEVD-CHO	190		B (single-protein format)	Test	Active	98.99
CHEMBL417149	Ac-DEVD-CHO	70,000		B (cell-based format)	Train	Inactive	72.97
CHEMBL421	Sulfasalazine	28,812		Eight experimental conditions	VS	Inactive	93.13
CHEMBL57267	Thioflavin T	762	TNF-alpha	F (assay format)	Train	Active	59.64
CHEMBL628	Pentoxifylline	85,000		Eight experimental conditions	VS	Inactive	80.74

^a^ Experimental IC_50_ values, which have been retrieved from the ChEMBL database. ^b^ Target protein against which the assay was carried out. ^c^ Experimental information associated with different assay protocols as depicted in Table 1, which also contains the eight experimental conditions *cj* under which the molecules were tested. ^d^ The notations “Train.” and “VS” stands for training and virtual screening, respectively. The molecules in the “Test” and “VS” sets were never used to build the mtc-QSAR-MLP model. ^e^ Annotating the molecules as active [*ACTi*(*cj*) = 1] or inactive [*ACTi*(*cj*) = −1] was realized by comparing the IC_50_ value of each molecule with the IC_50_ cutoffs (see Table 1). ^f^ Probability of belonging to a defined class (active or inactive) according to the observed value of *ACTi*(*cj*); the probabilities reported for sulfasalazine and pentoxifylline are average probabilities.

**Table 5:** Top 20 ranked agency-regulatory chemicals predicted by the mtc-QSAR-MLP model as dual inhibitors of caspase-1 and TNF-alpha.

Molecule ChEMBL ID	Name	Avg (Prob.%) ^a^
CHEMBL237500	Linagliptin	100.00
CHEMBL3138665	Euquinine	100.00
CHEMBL170	Quinine	100.00
CHEMBL553204	Icariin	100.00
CHEMBL4297455	AT-001	100.00
CHEMBL548228	Ethylhydrocupreine	100.00
CHEMBL2079611	Hydroquinine	100.00
CHEMBL2104401	Detorubicin	99.99
CHEMBL2106451	Galarubicin	99.99
CHEMBL485980	Bucladesine	99.99
CHEMBL3989596	Leurubicin	99.98
CHEMBL226335	Rutin	99.97
CHEMBL169896	OSI-7904	99.97
CHEMBL277062	Bromazepam	99.96
CHEMBL2107085	Tocladesine	99.95
CHEMBL1697854	Zorubicin	99.95
CHEMBL3793226	GSK-945237	99.93
CHEMBL298734	Lonafarnib	99.93
CHEMBL2110953	Nantradol	99.91
CHEMBL4210847	PF-00489791	99.90

**^a^** This is the probability of a molecule to be a dual inhibitor of caspase-1 and TNF-alpha; this probability is calculated as the average of eight probability values, each of them associated with each of the eight experimental conditions reported in the dataset used to build the mtc-QSAR-MLP model.

## Data Availability

All the chemical and biological (raw) data were retrieved from the public repository known as ChEMBL (Available online: https://www.ebi.ac.uk/chembl/, accessed on 4 September 2021).

## Supplementary Materials

The following are available online at https://www.mdpi.com/article/10.3390/biom11121832/s1. Supplementary Material S1: Topological indices, averages, and standard deviation values, Supplementary Material S2: *D*[*GTI*]*cj* descriptors, classification results, and local metrics, Supplementary Material S3: Applicability domain of the cases/molecules in the dataset used to build the mtc-QSAR-MLP model, Supplementary Material S4: Topological indices for the 8922 agency-regulated molecules, Supplementary Material S5: *D*[*GTI*]*cj* descriptors of the 8922 agency-regulated molecules, virtual screening results, and assessment of the applicability domain, Supplementary Material S6: Metrics FA(%) and S(TSAD).
